# Activation of the PI3K/AKT Pathway in Merkel Cell Carcinoma

**DOI:** 10.1371/journal.pone.0031255

**Published:** 2012-02-17

**Authors:** Christian Hafner, Roland Houben, Anne Baeurle, Cathrin Ritter, David Schrama, Michael Landthaler, Juergen C. Becker

**Affiliations:** 1 Department of Dermatology, University of Regensburg, Regensburg, Germany; 2 Department of Dermatology, University Hospital Würzburg, Würzburg, Germany; 3 Department of General Dermatology, Medical University Graz, Graz, Austria; The Moffitt Cancer Center & Research Institute, United States of America

## Abstract

Merkel cell carcinoma (MCC) is a highly aggressive skin cancer with an increasing incidence. The understanding of the molecular carcinogenesis of MCC is limited. Here, we scrutinized the PI3K/AKT pathway, one of the major pathways activated in human cancer, in MCC. Immunohistochemical analysis of 41 tumor tissues and 9 MCC cell lines revealed high levels of AKT phosphorylation at threonine 308 in 88% of samples. Notably, the AKT phosphorylation was not correlated with the presence or absence of the Merkel cell polyoma virus (MCV). Accordingly, knock-down of the large and small T antigen by shRNA in MCV positive MCC cells did not affect phosphorylation of AKT. We also analyzed 46 MCC samples for activating *PIK3CA* and *AKT1* mutations. Oncogenic *PIK3CA* mutations were found in 2/46 (4%) MCCs whereas mutations in exon 4 of *AKT1* were absent. MCC cell lines demonstrated a high sensitivity towards the PI3K inhibitor LY-294002. This finding together with our observation that the PI3K/AKT pathway is activated in the majority of human MCCs identifies PI3K/AKT as a potential new therapeutic target for MCC patients.

## Introduction

Merkel cell carcinoma (MCC) is a very aggressive malignant skin tumor. The disease typically affects elderly patients. It is preferentially localized in the chronically UV-exposed skin. The correlation between UV light and MCC is probably due to the immunosuppressive rather than the mutagenic effect of UV irradiation. In patients with immunosuppression, MCC may occur at a significantly younger age.

The pathogenesis of MCC is as yet not completely understood [1], but the recent demonstration that the Merkel cell polyoma virus (MCV) DNA is frequently present in MCC suggests a viral induced carcinogenesis [2], [3]. Despite the recent demonstration that MCV infected MCC cells require expression of the MCV encoded T antigens for proliferation and survival [4], little is known on cooperating oncogenic events. Previous studies found no evidence for mutations in classical oncogenes [5].

Still, high resolution comparative genomic hybridization revealed a number of chromosomal regions with gains and losses in MCC; the frequent loss of chromosome 10 where the tumor suppressor gene phosphatase and tensin homologue (PTEN) is encoded, suggests that aberrations of the PI3K/AKT pathway may be involved in the pathogenesis of MCC [6]. Moreover, while inactivating *PTEN* mutations are rare in MCC, the lack of PTEN protein expression is frequent observed in MCC [7].

The PI3K/AKT (phosphatidylinositol 3-kinase/v-akt murine thymoma viral oncogene homologue) pathway is a major signaling pathway downstream of many growth factor receptors and possibly the most frequently activated signaling pathway in human cancer [8]. Indeed, it has an important impact on apoptosis, proliferation, cell growth and malignant transformation. PI3K contributes to the signaling from receptor tyrosine kinases upon growth factor binding and generates the second messenger phosphatidylinositol-3,4,5-trisphosphate (PIP_3_). PTEN reverses this step. PIP_3_ induces downstream phosphorylation and activation of the survival kinase AKT1. Besides loss of PTEN, the PI3K/AKT pathway can be activated by oncogenic mutations. Somatic mutations in the *PIK3CA* gene, encoding for the α isoform of the p110 subunit of PI3K, have been identified in a wide variety of human tumors including benign skin tumors [9], [10]. Furthermore, an oncogenic hotspot mutation in the pleckstrin homology domain (PHD) of *AKT1* is present in several tumor entities, albeit at a lower frequency than *PIK3CA* mutations [11].

Here, we demonstrate PI3K/AKT pathway activation, which is independent of the presence of MCV, and oncogenic *PIK3CA* mutations in human MCC. Activating *PIK3CA* mutations appear to occur at a low frequency, indicating that additional mechanisms contribute to PI3K/AKT pathway activation in MCC.

## Materials and Methods

### Sample acquisition

Formalin-fixed paraffin embedded histologically proven MCC samples (primary tumors and metastases) were retrieved from histological files for the generation of a tissue microarray and for DNA isolation. Written, informed consent had been obtained from all patients to use tumor material not needed for histopathological diagnosis for further scientific workup; the study was performed according to the guidelines of the local ethics committee (Ethikkommission der Medizinischen Fakultät der Universität Würzburg; sequential study number 124/05) and the declaration of Helsinki. In addition, MCC cell lines were used. The cell lines WaGa, BroLi, HeRo and LoKe were derived from MCC patients of the Department of Dermatology, University of Würzburg [4], while UISO, [12] MCC13, [13] MCC26 [14], MKL-1 and MKL-2 [15] have been established in other laboratories. DNA was isolated from cell lines and formalin-fixed paraffin-embedded tissues containing at least 60–80% of tumor cells using standard protocols.

### Immunohistochemistry

Immunohistochemistry was performed using a tissue microarray for MCC, malignant melanoma and basal cell carcinoma. The staining followed standard protocols. The antibody was directed against phosphorylated AKT at threonine 308 (rabbit polyclonal (#38449), Abcam, Cambridge, UK) and was used at a dilution of 1∶200. The overall pAKT T308 staining intensity (not the frequency of positive tumor cells) was scored from 0 (negative), 1+ (weak), 2+ (strong), and 3+ (very strong) by two individual investigators (R.H. and J.C.B.). Each sample was represented in triplicate on the tissue microarray. In total, 41 samples (most of them not identical with the MCC used for genetic analyses; 14 primary tumors and 27 metastases) were evaluated on the MCC tissue microarray, as well as 67 melanomas (17 nodular, 17 acrolentiginous, 16 lentigo maligna melanoma, 17 melanoma metastases) and 45 basal cell carcinomas (20 nodular, 17 nodular and ulcerated, 2 nodular and pigmented, 6 not available).

### Knock down of the MCV LT antigen

The MCV positive MCC cell lines WaGa, BroLi, MKL-1 and MKL-2 were infected with the lentiviral shRNA vector KH1 encoding either a scrambled shRNA or a shRNA targeting the MCV T antigen mRNAs. Successful knock down of large and small T antigen in MCV positive MCC cells using this construct has been recently described [4]. Total cell lysates were harvested on day 5 following infection and analyzed by immunoblotting. T antigen knock down was confirmed using the Large T antigen (LT) specific antibody CM2B4 [16]. α-AKT and α-phospho-AKT (T308 and S473) antibodies were purchased from Cell Signaling (Danvers, MA, USA).

### PI3K/AKT inhibition

Cells were seeded in 96 well plates and the PI3K inhibitor was added at varying concentrations. After an incubation period of 24, 48 and 72 hours cellular metabolic activity was assessed by the MTS assay (CellTiter 96® AQueous One Solution Cell Proliferation assay, Promega Corporation, Madison, WI, USA). To this end, 10 µl of CellTiter 96® AQueous One Solution Reagent containing a tetrazolium compound (MTS) were added to each well and the cells were incubated for approximately 5 hours at 37°C. Metabolically active, viable cells convert MTS into a colored formazan product that was measured in a spectrophotometric microplate reader (Perkin-Elmer Inc., MA, USA) at 493 nm. Furthermore, the cellular DNA content was measured in ethanol fixed, propidium iodide stained cells by flow cytometry as described previously [4].

### Genetic analyses

Exons 9 and 20 of the *PIK3CA* gene were sequenced directly. These exons contain the majority of *PIK3CA* mutations yet found in human cancer. Samples which could not be sequenced successfully were analyzed by a recently described, more sensitive *PIK3CA* SNaPshot® assay in combination with a nested-PCR approach [17]. This assay covers the most important hotspot mutations at codons 542, 545 and 1047 of the *PIK3CA* gene. Furthermore, exon 4 of *AKT1* was sequenced directly, as this exon harbors the E17K hotspot mutation. In 8 MCC cell lines, exon 3 of *AKT3* was sequenced directly. Primer sequences and PCR conditions can be obtained from the authors. The presence of MCV DNA was assessed as described previously [2].

### Statistical analysis

After the data passed the Shapiro-Wilk normality test, statistical differences between two groups were evaluated by the t-test, and between more than two groups by the repeated measures ANOVA followed by the Dunnett's multiple post test. A *p* value≤0.05 was considered as significant.

## Results

### AKT activation in MCC

**Figure 1:**
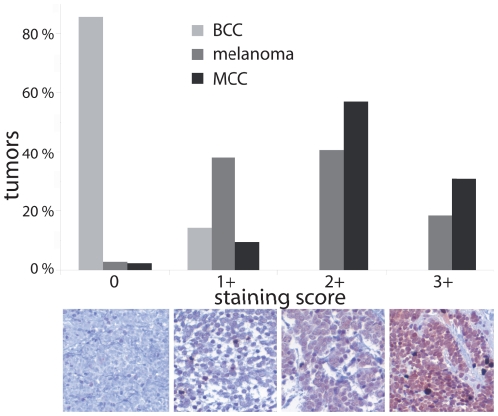
Activating phosphorylation of the AKT protein at position threonine 308 in Merkel cell carcinoma, malignant melanoma and basal cell carcinoma. The presence of AKT phosphorylated at T308 was analyzed by immunohistochemistry using a phospho-specific antibody on tissue micro arrays representing 41 MCCs (each in triplicates), 45 basal cell carcinomas (BCC) and 67 melanomas, respectively. Samples were scored from 0 (negative) to 3+ (strongly positive). The percentage of the samples for each expression score is indicated as bar graph and examples for the staining intensity in MCC are depicted below. In total, 88% of MCC samples showed strong (+2) or very strong (+3) staining for phospho-AKT T308, while 59% of melanoma samples were observed in these categories. Basal cell carcinoma showed only negative or weak AKT phosphorylation.

To investigate a possible activation of the PI3K/AKT pathway in MCC, immunohistochemical staining was performed for phospho-AKT threonine 308 (pAKT T308) taking advantage of a MCC tissue microarray. In total, 41 MCC samples (14 primary tumors and 27 metastases from a total of 26 patients) were evaluated. Expression of pAKT T308 was scored from 0 (negative) to 3+ (strongly positive). One MCC (2%) was categorized as negative (0), 4 (10%) as weak (1+), 23 (56%) as strong (2+) and 13 (32%) as very strong (3+) for their respective pAKT T308 expression (**Figure 1**). There were no significant differences between primary tumors and metastases regarding pAKT expression (mean expression scores of 2.36 (primary tumors) and 2.07 (metastases); *p* = 0.23). In addition, 8 MCC cell lines were stained for pAKT T308 (**Table 1**). Four cell lines revealed a strong (2+) and four MCC cell lines a very strong (3+) expression of pAKT T308. The immunohistochemical results obtained by analyzing both MCC tumor tissue and cell lines indicate that in the majority of MCC the PI3K/AKT pathway is activated.

**Table 1:** Analysis of *PIK3CA*, *AKT1* and pAKT T308 in Merkel cell carcinoma.

					*PIK3CA*		*AKT1*	IHC
no.	sex	age	type	MCV	Exon 9	Exon 20	Exon 4	pAKT T308
1	f	46	cell line (UISO)	−	wt	wt*	wt	2
2	f	80	cell line (MCC13)	−	wt	wt	wt	2
3	m	64	cell line (MCC26)	−	wt	wt	wt	2
4	m	26	cell line (MKL-1)	+	wt	wt	wt	3
5	m	55	cell line (BroLi)	+	wt	wt	wt	3
6	m	67	cell line (WaGa)	+	wt	wt	wt	2
7	f	73	cell line (HeRo)	+	wt	wt	wt	3
8	m	64	cell line (LoKe)	+	wt	wt	wt	3
9	m	83	metastasis	+	wt*	−	−	2
10	−	70	metastasis	−	wt*	−	−	−
11	−	44	metastasis	+	wt	wt	−	−
12	−	77	metastasis	+	wt*	−	−	−
13	−	45	metastasis	+	wt	wt	wt	−
14	f	80	metastasis	+	wt	wt	wt	−
15	m	66	metastasis	+	wt*	−	−	−
16	m	68	metastasis	+	wt*	−	−	3
17	m	−	metastasis	−	wt*	wt*	−	−
18	f	77	metastasis	+	wt*	wt*	−	2
19	f	72	metastasis	+	wt*	wt*	−	2
20	f	56	metastasis	+	wt*	wt*	−	−
21	f	68	primary tumor	+	wt	wt*	wt	−
22	m	46	metastasis	−	wt	wt*	wt	2
23	m	83	metastasis	+	**E545Q**	wt	wt	3
24	m	64	metastasis	+	wt	wt*	wt	−
25	m	77	metastasis	+	wt	wt	−	2
26	m	75	primary tumor	+	**E542K**	wt	wt	2
27	m	75	metastasis	+	wt*	wt*	−	−
28	m	79	primary tumor	+	wt*	−	−	2
29	−	58	metastasis	+	wt	wt*	−	−
30	−	73	metastasis	−	wt*	wt*	wt	−
31	f	80	metastasis	+	wt*	wt*	−	−
32	m	93	primary tumor	−	wt*	wt*	−	−
33	m	77	primary tumor	+	wt*	−	wt	−
34	f	65	primary tumor	+	wt	wt	wt	−
35	f	72	metastasis	+	wt*	wt*	−	−
36	m	82	primary tumor	+	wt	wt*	wt	−
37	m	80	primary tumor	+	wt*	wt*	−	−
38	f	89	primary tumor	+	wt	wt	wt	−
39	m	62	metastasis	+	wt	wt*	wt	−
40	m	55	primary tumor	+	wt	wt	wt	−
41	f	93	primary tumor	+	wt	wt	−	−
42	m	88	metastasis	+	wt	wt*	−	−
43	m	84	primary tumor	+	wt*	wt*	−	−
44	m	53	primary tumor	+	wt	wt*	−	−
45	m	49	primary tumor	+	wt*	wt*	−	−
46	m	82	metastasis	+	wt*	wt*	wt	−

Age, age at the time of diagnosis; m, male; f, female; MCV, Merkel cell polyoma virus (the MCV status was assessed as described previously [2]); wt, wild-type; wt*, these samples could not be sequenced directly, but were analyzed by a modified SNaPshot® assay; −, not available; IHC pAKT T308, immunohistochemistry for phospho-AKT threonine 308 scored from 0 (negative) to 3+ (very strong) staining intensity; it has to be noted that only 9 samples used for mutation analysis were also present on the tissue microarray.

To compare the level of AKT activation in MCC with other skin cancers, we additionally performed immunohistochemical staining for pAKT T308 in 67 malignant melanomas, a tumor with established frequent AKT pathway activation [18], [19], and 45 basal cell carcinomas which have been shown to be characterized by only low levels of phospho-AKT [20], [21]. In line with these pre-published data, basal cell carcinomas stained negative or very weakly for pAKT, while melanomas revealed a higher phosphorylation (**Figure 1**). Malignant melanoma showed lower levels of AKT phosphorylation than MCC with 59% of samples categorized as strong (+2) or very strong (+3), compared with 88% of MCC samples. However, the comparability of the different tumor entities might be diminished by different proportions of primary versus metastatic tissues.

### AKT activation is independent of MCV

**Figure 2:**
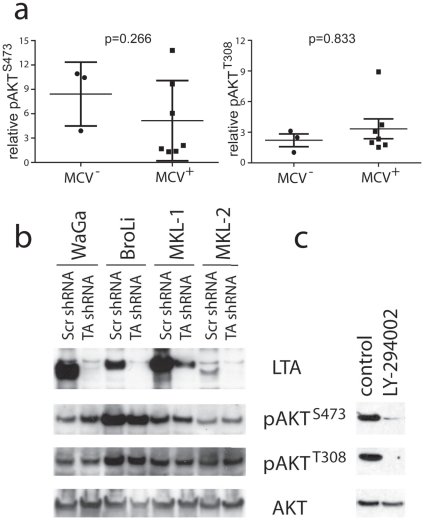
Merkel cell polyomavirus T antigens do not affect AKT phosphorylation in Merkel cell carcinoma. **a)** Total cell lysates of 7 MCV positive and 3 MCV negative MCC cell lines were subjected to Western blot analysis applying antibodies to pAKT^T308^, pAKT^S473^ and tubulin. Signal intensity was quantified using the imageJ software and the values normalized p-values according to the Mann-Whitney are indicated. **b)** The indicated cell lines were infected with the lentiviral shRNA vector KH1 encoding GFP and either a shRNA targeting all MCV TA mRNAs or a scrambled shRNA; Infection rates as determined by GFP flow cytometry analysis were 98% for WaGa, 94% for BroLi, 90% for MKL-1 and 96% for MKL-2. Total cell lysates harvested on day 5 following infection were then analyzed by immunoblotting for expression of large T antigen (LTA) and AKT and for the presence of AKT phosphorylated at T308 or S473. The variations in molecular size of the LTA proteins in the different cell lines are due to different stop codon mutations truncating the C-terminal part of the protein. **c)** Treatment with the PI-3 kinase inhibitor LY-294002 demonstrated inhibition of AKT phosphorylation at the phosphorylation sites T308 and S473 by blocking the upstream kinase.

Given the recent discovery that most MCCs are characterized by the integration of a polyoma virus, i.e. the Merkel cell polyomavirus (MCV), it is important to note that the oncogenic proteins encoded by polyomaviruses have been implicated in the activation of the PI3K/AKT pathway. For example, SV40 small T antigen inhibits the protein phosphatase 2A (PP2A) which dephosphorylates AKT on both activating phosphorylation sites [22]. Consequently, we addressed the possible contribution of MCV T antigens to AKT activation in MCC cells. To this end, AKT phosphorylation was measured by Western blot analysis in seven MCV positive and three MCV negative cell lines. Quantification of the western blot signals, however, demonstrated lack of any significant correlation between pAKT and MCV status (**Figure 2 a**). Furthermore, a possible functional role of the MCV T antigens on AKT activation in MCC was tested by silencing T antigen expression in four MCV positive MCC cell lines by infection with a lentiviral shRNA construct targeting Large as well as small T antigen (LT and sT) mRNA; LT and sT mRNA are derived by alternative splicing from the same genomic locus and share common sequences in exon 1 [3]. T antigen knock down did not affect phosphorylation of AKT at T308 and S473 in any of the tested cell lines (**Figure 2 b**). In contrast, treatment with the PI-3 kinase inhibitor LY-294002 demonstrated that AKT phosphorylation at these two sides can be inhibited by blocking the upstream kinase (**Figure 2 c**).

In accordance with the observations in the cell lines, the expression level of pAKT T308 *in situ* did not correlate with the MCV status (i.e. immunohistochemical mean scores of 2.11 and 2.21 for MCV negative (n = 9) and positive (n = 29) samples, respectively; *p* = 0.74). These lines of evidence strongly suggest that the MCV T antigens are not critical for AKT pathway activation in MCC cells, although it cannot be excluded that the difference between MCV negative and positive samples would reach significance with a higher number of samples.

### *PIK3CA* mutations in MCC

**Figure 3:**
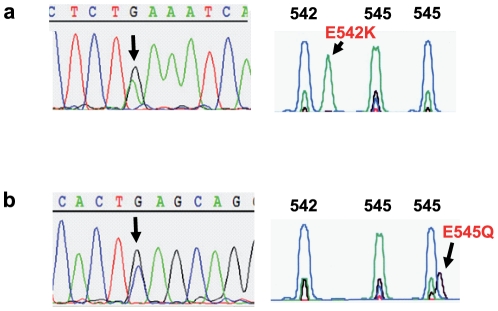
*PIK3CA* hotspot mutations in Merkel cell carcinoma. (a) The heterozygous p.E542K (c.G1624A) *PIK3CA* hotspot mutation was detected in sample no. 27 by direct sequencing (left) and a *PIK3CA* SNaPshot® assay (right) covering the most frequent hotspot *PIK3CA* mutations. The number of the wildtype codons is indicated above the peaks in the SNaPshot® assay. In brief, the SNaPshot® assay comprises a multiplex PCR for exons 9 and 20 of *PIK3CA*, followed by extension of 4 primers specific for the most frequent *PIK3CA* hotspot mutation loci. Because fluorescent dideoxynucleotides are used for this primer extension step, only one peak appears at the base position with the potential mutation. The color of the peak allows discrimination of wildtype and mutated alleles. (b) The heterozygous p.E545Q (c.G1633C) *PIK3CA* hotspot mutation was detected in sample no. 24 by direct sequencing (left) and a *PIK3CA* SNaPshot® assay (right) covering the most frequent hotspot *PIK3CA* mutations. The number of the respective wildtype codon is indicated above the peaks in the SNaPshot® assay.

In many cancers, PI3K/AKT pathway activation is mediated by oncogenic mutations in *PIK3CA* and *AKT1* genes. Thus, we tested both the MCC tissue samples as well as the MCC cell lines for *PIK3CA* and *AKT1* hotspot mutations. The characteristics of the patients and samples are given in **Table 1**. In total, 46 MCC samples (14 primary tumors, 24 metastases and 8 cell lines) were analyzed. Heterozygous *PIK3CA* mutations were identified in two out of 46 samples (4%) (**Figure 3**). Both mutations (E542K and E545Q) are localized in exon 9 of *PIK3CA*, which encodes the helical domain, and in both cases the tumors harbored MCV DNA. Both mutations were tested by an independent second PCR and additionally by a modified *PIK3CA* SNaPshot® assay. The detected mutations were independently confirmed by these alternative methods. DNA isolated from peripheral blood lymphocytes revealed a wildtype status in both cases, thus confirming the somatic nature of the mutations. Subsequently, additional samples of the two patients were tested. The patient with the E542K mutation in the primary tumor revealed the same mutation in two metastases of the temple and the parotid gland. The E545Q mutation found in a metastasis of the second patient was detected in two of three further metastases on the head. These results suggest that in both cases the mutation occurred before metastatic tumor spread.

The two MCC samples harboring a *PIK3CA* mutation were also present on the tissue microarray. The sample with the E542K mutation revealed a strong (2+) and the one with the E545Q mutation a very strong (3+) pAKT T308 protein expression (**Table 1**). Increased AKT phosphorylation can also be caused by defined *AKT* mutations. The E17K *AKT1* mutation causes a pathological localization of AKT1 to the plasma membrane and thereby stimulates downstream signaling proteins [23]. Sequencing of exon 4 of *AKT1*, containing the E17K hotspot locus, was possible for 24 samples, but did not reveal any *AKT1* mutation in MCC. In addition, exon 3 of *AKT3* harbouring the E17 hotspot locus was sequenced in 8 MCC cell lines (UiSo, MCC13, MCC26, MKL-1, LoKe, WaGa, HeRo, MaTi, BroLi), but did not reveal any mutations.

### MCC cells are sensitive to PI3K inhibition

**Figure 4:**
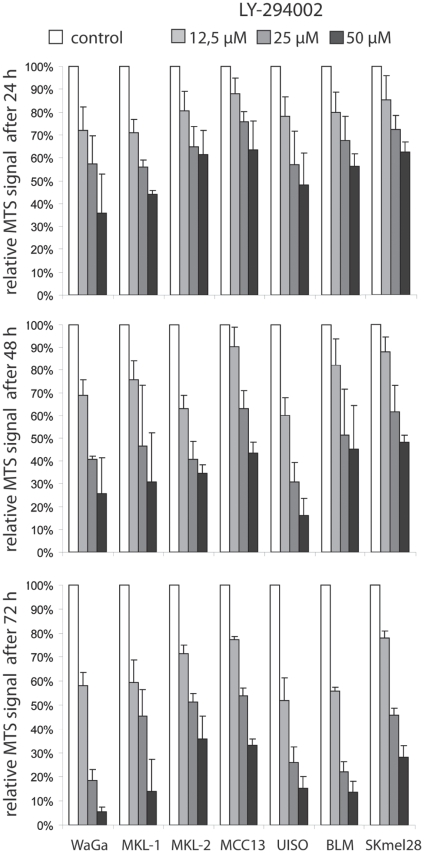
MCC cell lines are sensitive to the PI3K inhibitor LY-294002. The indicated MCC and melanoma cell lines were incubated with LY-294002 at three different concentrations. After the indicated time period the cells were subjected to the MTS assay in triplicates. The reduction in extinction relative to the DMSO (solvent of LY-294002) controls is depicted. The graphs represent mean values (± standard deviation) of at least three independent experiments.

We analyzed whether inhibition of the PI3K/AKT pathway would impact the viability and growth of MCC cells. To this end, we incubated 5 MCC cell lines (WaGa, MKI-1, MKI-2, MCC13, UISO) for 24, 48 and 72 hours with the PI3K inhibitor LY-294002 at different concentrations (range 12.5–50 µM) and subsequently analyzed the cells using the MTS assay. In addition, since inhibition of the AKT pathway is regarded as a reasonable therapeutic option in melanoma [24], [25], two melanoma cell lines carrying known PI3K/AKT pathway activating mutations served as positive controls; Skmel-28 was described to have a *PTEN* mutation [26] while sequencing of the *NRAS* gene in BLM cells revealed a Q61R mutation (data not shown). LY-294002 reduced the metabolic activity for all 6 MCC cell lines in a time and dose dependent manner between 10–95% (**Figure 4**). Indeed, at all time points analyzed the means were significantly different (p<0.0001; repeated measures ANOVA); Dunnett's multiple post test revealed significantly inhibition by each inhibitor concentration compared to control (p<0.05). Interestingly, the MCC cell lines demonstrated in response to the PI3K inhibitor at least an equal reduction in MTS signal as the melanoma cell lines, which are proliferating much faster than most of the MCC cell lines (**Table 2**). The MTS assay does not distinguish between apoptosis and cell cycle arrest. Therefore, we performed DNA staining after 40 hours of LY-294002 (25 µM) treatment. Only WaGa cells displayed a strong increase in sub-G1 cells at this time point while all other cell lines - although dead cells were increased in all cases in the presence of LY-294002 – showed only a moderate apoptotic response (**Table 2**). Cell cycle arrest induced by the PI-3 kinase inhibitor was also observed in all cell lines but the measured decrease in S-phase cells was quite moderate in many cases (**Table 2**). Although we know from previous experiments that a cell cycle arrest is difficult to demonstrate in the slowly cycling MCC cells [4] it is likely that not only reduced cell numbers but also reduced metabolic activity per cell may contribute to the effects in the MTS assay. Nevertheless, analysis of the cellular DNA content suggests that both, cell cycle arrest as well as apoptosis induction contribute to inhibition by LY294002 in MCC cells.

**Table 2:** Response of Merkel cell carcinoma and melanoma cell lines to LY-294002.

	doubling time (days)	IC 50_LY-294002_ [µM]			cells in S-phase [%]		sub-G1 cells [%]	
		24 hours	48 hours	72 hours	control	LY-294002	control	LY-294002
**WaGa** *	3	32,5	23,1	12,0	6,8	4,8	4,5	31,1
**MKL-1** *	3	38,3	27,6	18,4	9,4	5,8	2,1	4,8
**MKL-2** *	4	64,3	26,1	30,1	9,3	3,9	19,5	27,8
**MCC13**	1	74,3	41,6	30,7	12,5	10,3	1	4,3
**UISO**	2	43,0	16,8	14,4	14,7	8	5,2	8,8
**BLM**	1	57,2	38,8	13,8	11	5,8	1,7	2,9
**Skmel28**	1	70,9	45,7	26,4	15,1	3,6	0,6	1,3

Doubling times were roughly estimated from the necessary split ratios during culture. IC50 values for the inhibition by LY-294002 were calculated from the MTS assay date depicted in Figure 4 assuming an exponential relation. The percentage of S-phase and sub-G1 cells were estimated by flow cytometry analysis of propidium iodide stained cells following 40 hours in the presence of 25 µM LY-294002.

*MCV positive cell lines.

## Discussion

*PIK3CA* mutations have been already identified in a broad range of human cancers at varying frequencies, including liver (36%), breast (26%), colon (25%), urothelial (13%), ovarian (9%), gastric (7%), brain (6%), and lung cancer (2%) as well as leukaemia (1%) [9], [27]. The present study adds Merkel cell carcinoma to this list of human cancers harboring *PIK3CA* mutations, although in MCC these mutations occur obviously at a low frequency. Both mutations are localized in the helical domain, whereas in most other cancers, mutations in the kinase domain, i.e. in exon 20, are more common (www.sanger.ac.uk/genetics/CGP/cosmic/). Nevertheless, the E542K mutation is one of the most frequent *PIK3CA* hotspot mutations and results in a strong activation of PI3K [28]. The E545Q missense mutation is less frequent but has been described in breast, anaplastic thyroid, ovary, and esophageal cancer [29].

To the best of our knowledge, this is the first report of oncogenic mutations in human MCC. Notably, the analysis of several other oncogenes including *HRAS*, *KRAS*, *NRAS*, *BRAF*, *C-KIT*, and genes of the Wnt pathway did not show any mutations in human MCC [5], [30], [31], [32], [33]. Indeed, all of the previously reported mutations in MCC were restricted to tumor suppressor genes. *PTEN* mutations were observed at a very low frequency, although loss of heterozygosity at the *PTEN* locus at chromosome 10q seems to be a frequent event [34]. Similarly, mutations in other tumor suppressor genes such as *p53*, *p73* and *CDKN2A* have been reported, however, only in a very small fraction of MCCs.

Immunohistochemical analysis of the two MCC tumors carrying activating *PIK3CA* mutations demonstrated a strong AKT phosphorylation; this observation is in line with the fact that these mutations contribute to an activation of the PI3K/AKT signaling pathway. However, the two tumors harboring the activating *PIK3CA* mutations were not exceptional with respect to AKT pathway activity. In fact, 88% of a series of 41 MCC tissues were classified in the same categories (strong or very strong staining for pAKT T308) suggesting alternative mechanisms (e.g., PTEN alterations) of AKT pathway activation in the majority of cases.

Merkel cell polyomavirus has been recently identified as a widespread virus which upon integration into the genome of MCC precursor cells and acquisition of truncating mutations in the viral large T antigen is likely to contribute to MCC development and progression [3], [16], [35]. In this respect we recently demonstrated that MCV infected MCC cells require expression of the MCV T antigens for proliferation and survival [4]. MCV genomes encode for the presumably oncogenic large and small T antigens; for the respective homologs encoded by SV40 it has been demonstrated that they can activate the AKT pathway. While for small T this happens via inhibition of the protein phosphatase 2A [22], large T antigen activates the AKT pathway through its interaction with the insulin receptor substrate 1 [36]. Surprisingly, however, shRNA knock down of both MCV T antigens in infected MCC cell lines did not affect AKT phosphorylation and the lack of correlation between MCV status and AKT phosphorylation in the tumor samples also suggests that the MCV viral T antigens do not contribute to AKT pathway activation in MCC cells, although the significance of the latter observation is impaired by the low number of MCV negative samples. Thus, the presence of the viral proteins seems neither to be sufficient nor necessary for AKT pathway activation in MCC. A very recent report demonstrating that transformation of rodent fibroblasts by MCV small T antigen is independent of PP2A binding further supports that MCV T antigens function different than the related SV40 oncoproteins and that the AKT pathway is not a critical target of MCV T antigens [37]. Future studies are warranted to elucidate the molecular mechanisms for AKT pathway activation in MCC.

Since activation of the PI3K/AKT signaling pathway represents one of the most frequent events in human cancer, specific inhibitors of PI3K, AKT and additional components of the PI3K/AKT signaling pathway are currently tested in preclinical and clinical trials [8]. Notably, MCC showed a significantly higher AKT phosphorylation than malignant melanoma in our study. In melanoma, phosphorylation of AKT and activation of the PI3K/AKT signaling pathway is a well known feature. A previous study identified AKT phosphorylation in 66% of melanoma samples [18], congruent with the results observed in this study. Consequently, inhibition of the PI3K/AKT pathway by specific inhibitors has evolved as a promising treatment strategy for malignant melanoma [24], [25]. In our study, MCC cells showing strong activation of the PI3K/AKT pathway were sensitive to the PI3K inhibitor LY-294002 *in vitro* although we cannot exclude that off target effects may contribute to the observed inhibition.

Since metastasized MCC is a very aggressive tumor with poor prognosis and very limited therapeutic options, the presented observations are opening the avenue for targeting the activated PI3K/AKT pathway as an interesting new option for patients suffering from advanced MCC.
